# One-health approach on the future application of snails: a focus on snail-transmitted parasitic diseases

**DOI:** 10.1007/s00436-023-08021-z

**Published:** 2023-12-12

**Authors:** Chet Raj Pathak, Himal Luitel, Kjersti Selstad Utaaker, Prabhat Khanal

**Affiliations:** 1 Faculty of Animal Science, Veterinary Science and Fisheries, Agriculture and Forestry University, Rampur, Nepal; 2 Animal Science, Production and Welfare Division, Faculty of Biosciences and Aquaculture, Nord University, Skolegata 22, 7713 Steinkjer, Norway; 3 Center for Biotechnology, Agriculture and Forestry University, Rampur, Nepal

**Keywords:** Intermediate host, One-health, Snails, Safety, Zoonotic parasitic diseases

## Abstract

Snails are fascinating molluscs with unique morphological and physiological adaptive features to cope with various environments. They have traditionally been utilized as food and feed sources in many regions of the world. The future exploitation of alternative nutrient sources, like snails, is likely to increase further. Snails, however, also serve as an intermediate host for several zoonotic parasites. A category of parasitic infections, known as snail-transmitted parasitic diseases (STPDs), is harmful to humans and animals and is mainly driven by various trematodes, cestodes, and nematodes. The environment plays a crucial role in transmitting these parasites, as suitable habitats and conditions can facilitate their growth and proliferation in snails. In light of diverse environmental settings and biologically categorized snail species, this review evaluates the dynamics of significant STPDs of zoological importance. Additionally, possible diagnostic approaches for the prevention of STPDs are highlighted. One-health measures must be considered when employing snails as an alternative food or feed source to ensure the safety of snail-based products and prevent any adverse effects on humans, animals, and the environment.

## Introduction

Molluscs, such as snails, slugs, and mussels, are a distinct group of spineless animals and play vital roles in the ecosystem (Fortunato 2015; Rosenberg 2014; Vaughn and Hakenkamp 2001). The snail species have been used as a food source in different regions worldwide (Hill et al. 2015; Ngenwi et al. 2010). Many production animals, such as poultry and fish, naturally feed on snails (Amobi et al. 2019; Pertiwi and Saputri 2020). In light of finding novel animal feed ingredients to ensure future food security, snails have been recognized as a potential alternative feed source for production animals that can undergo commercial-scale production (Adeyeye et al. 2020; Munonye and Moses 2019). Snails are considered a valuable supplement to conventional feedstuffs because they contain high levels of protein, calcium, and other essential nutrients (Wright et al. 2018) (Table 1). In addition, snail farming can be operated under relatively low-cost animal husbandry practices that can be profitable in areas where snails are abundant (Mvodo Meyo et al. 2021). Snails have several applications in multiple sectors, such as agriculture, medicine, the food industry, cosmetics, biotechnology, and environmental monitoring, due to their unique biological characteristics and their ability to adapt to changing environments (Akan et al. 2020; Cilia and Fratini 2018; Wang et al. 2020; Yuan et al. 2019) (Table 2). While having a valuable component for various applications, it is noteworthy that they act as agents for serious parasitic disease transmission.

**Table 1:** Primary nutrient contents of different snail species (dry matter, DM)

Snail species	Items	CHO (%DM)	TP (%DM)	CF (%DM)	Ash (%DM)	Fat %	Ca	Fe	References
*Achatina achatina*	Fresh snail meat	24.38	63.46	2.88	1.97	2.85	201.36	0.64	Uboh et al. (2014)
*Archachatina marginata*	Fresh snail meat	22.53	63.46	3.01	2.08	2.40	199.26	0.64	Uboh et al. (2014)
*Achatina fulica*	-	-	83.13	-	8.90	8.70	-	-	Ghosh et al. (2016)

*CHO*, carbohydrates; *TP*, total protein; *CF*, crude fiber; *Ca*, calcium (mg/100g); *Fe*, iron (mg/100g)

**Table 2:** Various applications of different snail species

Snail species	Items	Uses/properties	References
*Archachatina marginata*	Snail meat	Food industry	Djikeng et al. (2022)
*Lymnaea stagnalis*	Crude extract, mucus, glycans, polypeptides	Cosmetic, pharmaceutical (treatment of wart, cancer, dementia, and Alzheimer’s)	Dhiman and Pant (2021)
*Lissachatina fulica*	The mucus of the mantle collar, foot	Antioxidant, antibacterial, and anti-tyrosinase activities	Noothuan et al. (2021)
*Helix aspersa muller*	Snail mucus	Against gastric or peptic ulcers, antibacterial	Di Filippo et al. (2022), Gugliandolo et al. (2021)
*Achatina fulica*	Snail mucus	Anti-cancer	Edison et al. (2021)
Snails	Secretions	Biotechnology: dual properties on cancer	Chang et al. (2020)
*Pomacea canaliculata*	Snail body	Environmental monitoring: bioaccumulation of heavy metal analysis	Dummee et al. (2012)

Snail-transmitted parasitic diseases (STPDs) are a significant public health concern in many parts of the world, particularly in tropical and sub-tropical regions (Lu et al. 2018). Snails serve as intermediate hosts in the transmission of various parasites, primarily three classes of helminths: trematodes, nematodes, and cestodes (Chakraborty and Joy 2020; Lopatek et al. 2022). Snails act as single intermediate hosts for certain parasites, such as *Angiostrongylus* and *Schistosoma mansoni*, whereas in others, e.g., *Clonorchis sinensis*, *Paragonimus westermani*, *Fasciolopsis buski*, and *Fasciola hepatica*, snails can be only first intermediate hosts and then the parasitic larva invade other second intermediate hosts like fish, crab, or encysted in the aquatic vegetations for more asexual reproductions (Ansari et al. 2021; Mehmood et al. 2017). After invading snails, the parasites go through some developmental stages. For example, in the case of most trematodes, miracidium penetrates the snails, develops further into sporocyst and redia, and then releases cercaria from the snails (Galaktionov and Dobrovolskij 2013). Then, the cercaria encysts and develops into metacercaria that can enter into livestock and human bodies through the consumption of feed, fodder, food, and water, or cercaria in the case of schistosomes can directly penetrate skins through contaminated water bodies (Lv et al. 2018). It is important to note that not all snail species are suitable for use as animal feed, and it is necessary to choose snail species that are safe, nutritious, and affordable for the intended animal food or feed applications. In this context, understanding the biology of snails and their interactions with parasites and pathogens is crucial for ensuring their safe and sustainable use within the food and feed sector (Colgan 2020; Imathiu 2020; Pissia et al. 2021; Ponder et al. 2019a).

## The biology of snails in relation to parasitic disease transmission

Snails are essential hosts for many parasites, including those that cause significant morbidity and mortality in humans and animals. Understanding the physiological processes of snails is crucial to getting insights into the complex interactions between parasites and snail hosts and in developing effective prevention and control measures and management of snail-transmitted parasitic diseases (Lu et al. 2018; Malek 2018; Ponder et al. 2019b).

### Characteristics of snail species

**Fig. 1:**
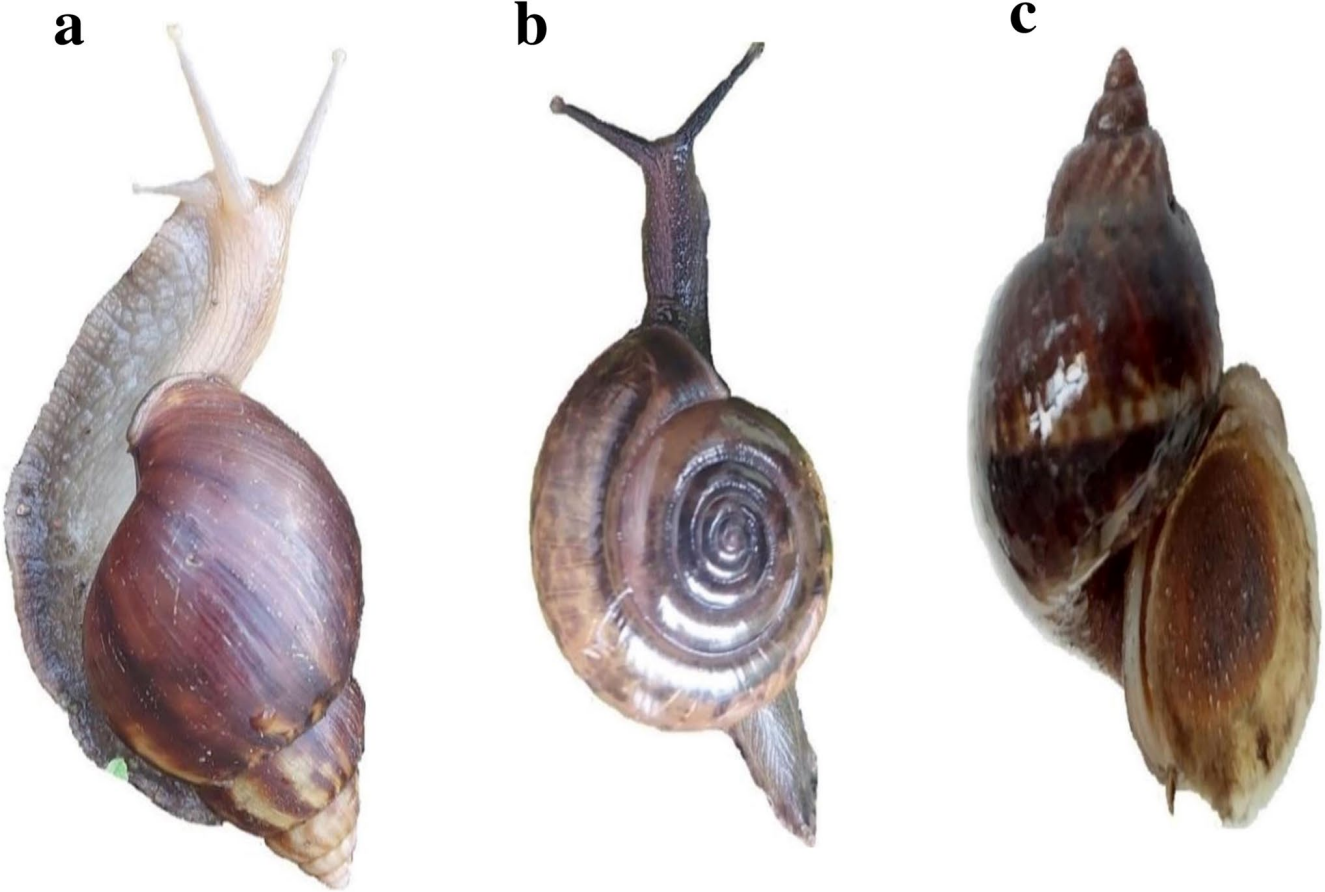
Three different snail species were collected from Rampur, Chitwan, Nepal (latitude: 27.658734, longitude: 84.35105; 4^th^ September 2022; 28.7 °C). The morphological identification and comparison of published databases (Glöer and Bössneck 2013; Raheem et al. 2010) may suggest the following species: **a** *Lissachatina* spp. (Achatinidae), **b** *Gyraulus* spp. (Planorbidae), and **c** *Radix* spp. (Lymnaeidae)

Snails are invertebrates belonging to the phylum, Mollusca, which is the second-largest animal group and is quite diverse (Ponder et al. 2019b). Under this phylum, gastropods are the largest, highly diversified class of molluscs consisting of snails and slugs present in saltwater, freshwater, and land. They are characterized by a single spiral shell and a foot for movement (Fig. 1) (Bouchet et al. 2017). Snails are further categorized into multiple orders based on certain morphological features and habitats, each containing several families (Table 3). The families including Achatinidae, Bradybaenidae, Camaenidae, Cyclophoridae, Helicarionidae, Subulinidae, and Succineidae belong to land snails, whereas the freshwater snails include Ampullariidae, Ancyclidae, Acroloxidae, Bithyniidae, Physidae, Planorbidae, Lymnaeidae, Viviparidae, Thiaridae, and Pomatiopsidae (Bouchet et al. 2017; Saadi et al. 2020; Soldatenko and Petrov 2019). Both land and water snail species are susceptible to being infected with at least one parasite during the life cycle from their surrounding environments, and the parasite can surpass the snails’ structural and biochemical barriers. However, the nature or species of the parasite infecting them depends on the body of snails and their physiological status, along with feeding and habitat diversity.

**Table 3:** Classification of important parasite-transmitting snail species

Subclass/order	Family	Snail species	Infecting parasites species	References
Pulmonata/Stylommatophora (Land snails)	Achatinidae	*Achatina* sp.	**AC**	Brozzo et al. (2020), Ding et al. (2016), Horsáková et al. (2020) Federspiel et al. (2020), Segeritz et al. (2021), Turck et al. (2022)
	Bradybaenidae	*Bradybaena* sp., *Euhadra* sp., *Plectotropis* sp.		
	Camaenidae	*Satsuma* sp., *Camaena* sp.		
	Cyclophoridae	*Pupin* sp.		
	Helicarionidae	*Parnarion* sp.		
	Subulinidae	*Allopeas* sp., *Opeas* sp., *Subulina* sp.		
	Succineidae	*Succinea* sp.		
Pulmonata/Hygrophila (Freshwater snails)	Ampullariidae	*Pila* sp., *Pomacea* sp.		
	Ancyclidae	*Ferrissia* sp.		
	Bithyniidae	*Alocinma* sp., *Bithynia* sp., *Parafossarulus* sp.	**CS**, **OF**, **OV**	Naveen and Singh (2019), Ovando and Marchi (2021), Palasio et al. (2018), Saadi et al. (2020), Saito (2022), Schultz et al. (2018); Adema et al. (2017), Anderson and Enabulele (2021), Hailegebriel et al. (2020), Llanwarne and Helmby (2021), Rey et al. (2021), Siles-Lucas et al. (2021)
	Physidae	*Physa* sp.	**AC**	
	Planorbidae	*Biomphalaria* sp.	**SM**, **AC**	
		*Bulinus* sp.	**SH**, **SI**	
		*Gyraulus* sp., *Hippeutis* sp.	**AC**, **FB**	
		*Indoplanorbis* sp.	**AC**	
		*Lanistes* sp.	**AC**, **SH**	
		*Planorbarius* sp.	**SH**	
		*Segmentina* sp.	**AC**, **CS**	
Heterobrachia/Basomatophora (Freshwater snail)	Lymnaeidae	*Fossaria* sp.	**FH**	Ansari et al. (2021), Caldeira et al. (2016), Jackiewicz (2020), Swart et al. (2020); Hu et al. (2021); Lalor et al. (2021)
		*Galba* sp., *Lymnaea* sp.	**FH**, **AC**	
		*Omphiscola* sp., *Pseudosuccinea* sp.	**FH**	
		*Radix* sp.	**FH**, **AC**	
		*Stagnicola* sp.	**FH**	
Prosobrachia/Architaenioglossa (Freshwater snails)	Viviparidae	*Bellamya* sp., *Cipangopaludina* sp., *Filopaludina* sp., *Sinotaia* sp.	**AC**	Dewi et al. (2017), Jin et al. (2022), Lydeard and Cummings (2019), Uehara et al. (2021)
Prosobrachia/Caenogastropoda (Freshwater snails)	Pomatiopsidae	*Neotricula* sp.	**SMe**	Haase et al. (2021), Lee et al. (2019), Li et al. (2021), Limpanont et al. (2020)
		*Oncomelania* sp.	**AC**, **SJ**	
		*Robertsiella* sp.	**SMa**	
Prosobrachia/Neotaenioglossa (Sea water snails)	Buccinidae	*Clea* sp.	**AC**	Squires (2022), Yamazaki et al. (2018), Zhang and Zhang (2018)
	Thiaridae	*Melanoides* sp.	**CS**, **PW**, **SH**	Lopes et al. (2021);, Wiggering et al. (2019); Blair (2022), Paller et al. (2021)
		*Tarebia* sp.	**PW**	

*AC*, *Angiostrongylus cantonensis*; *CS*, *Clonorchis sinensis*; *FB*, *Fasciolopsis buski*; *FH*, *Fasciola hepatica*; *OF*, *Opisthorchis felineus*; *OV*, *Opisthorchis viverrini*; *PW*, *Paragonimus westermani*; *SM*, *Schistosoma mansoni*; *SMa*, *Schistosoma malayensis*; *SH*, *Schistosoma haematobium*; *SMe*, *Schistosoma mekongi*; *SJ*, *Schistosoma japonicum*

## Anatomical and physiological features of snails

**Fig. 2:**
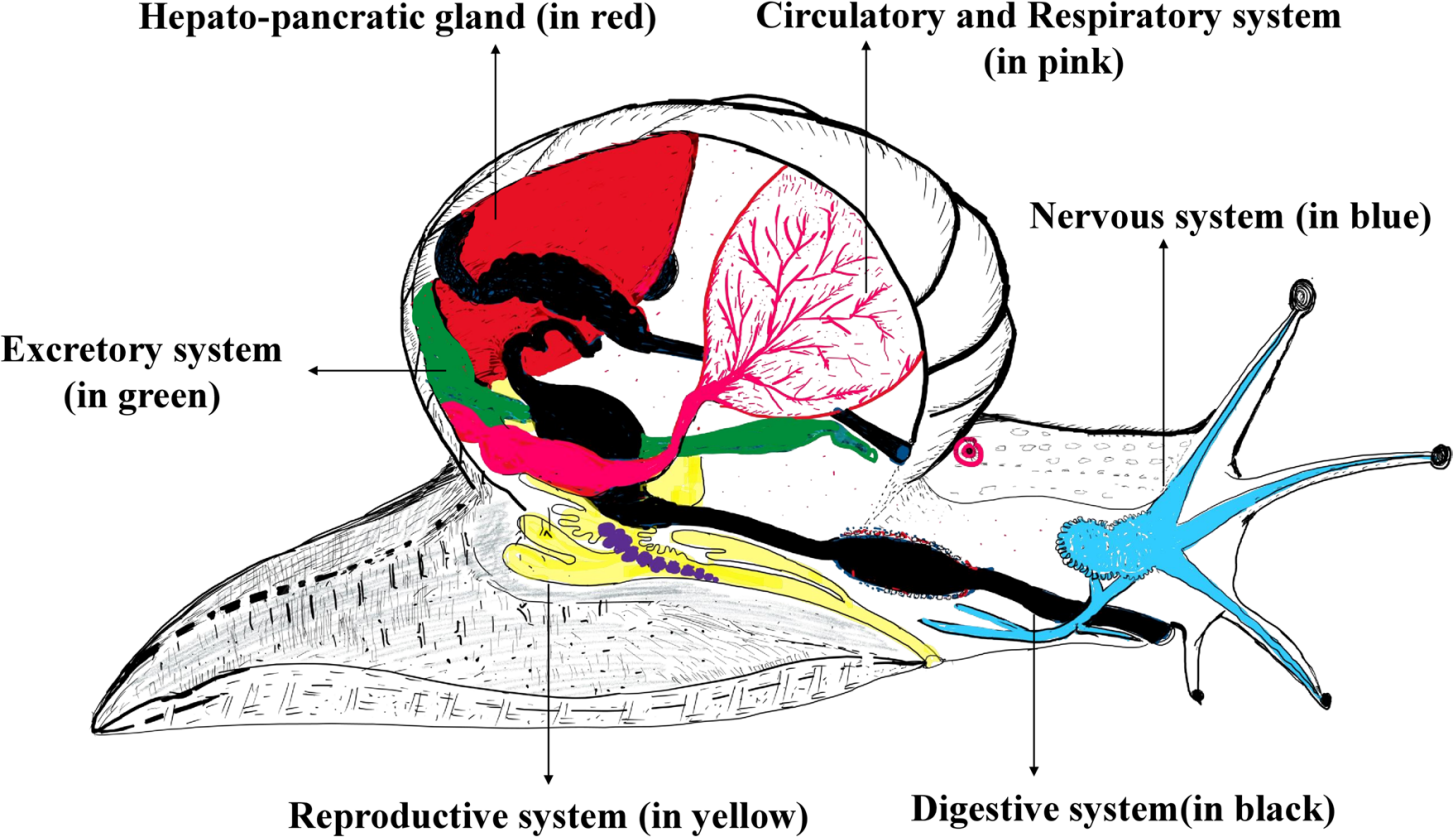
The major physiological systems of the snail. Nervous system in blue, circulatory and respiratory systems in pink, excretory system in green, reproductive system in yellow, digestive system in black, and digestive glands in red color. Adapted and modified from published literature (Ponder et al. 2019b)

The visceral mass is the main body cavity of the snail that contains digestive, respiratory, and reproductive organs (Fig. 2). The viscera are protected and supported by a species-specific shell containing calcium carbonate (Larsson et al. 2020; Parveen et al. 2020). The one pair of snail’s tentacles helps in sensory perception during the retraction of the head region (Brenzinger et al. 2021). The penetration of the first larval stage (miracidium) of *Schistosoma* species occurs from the outer surface of the head, foot, and tentacles, near the buccal mass esophagus, whereas the *Fasciola* species penetrates the mantle collar and anterior wall of the pulmonary sac (Malek 2018). The anatomical features and physiological activities of snails are crucial for the development, proliferation, and spread of pathogens in the body, as discussed below.

### The digestive system of snails

Parasites can induce physiological changes and pathology in their hosts, affecting metabolism, immune response, growth, development, and (Marcogliese). In natural environments and also in experimental investigations, fecundity

**Fig. 3:**
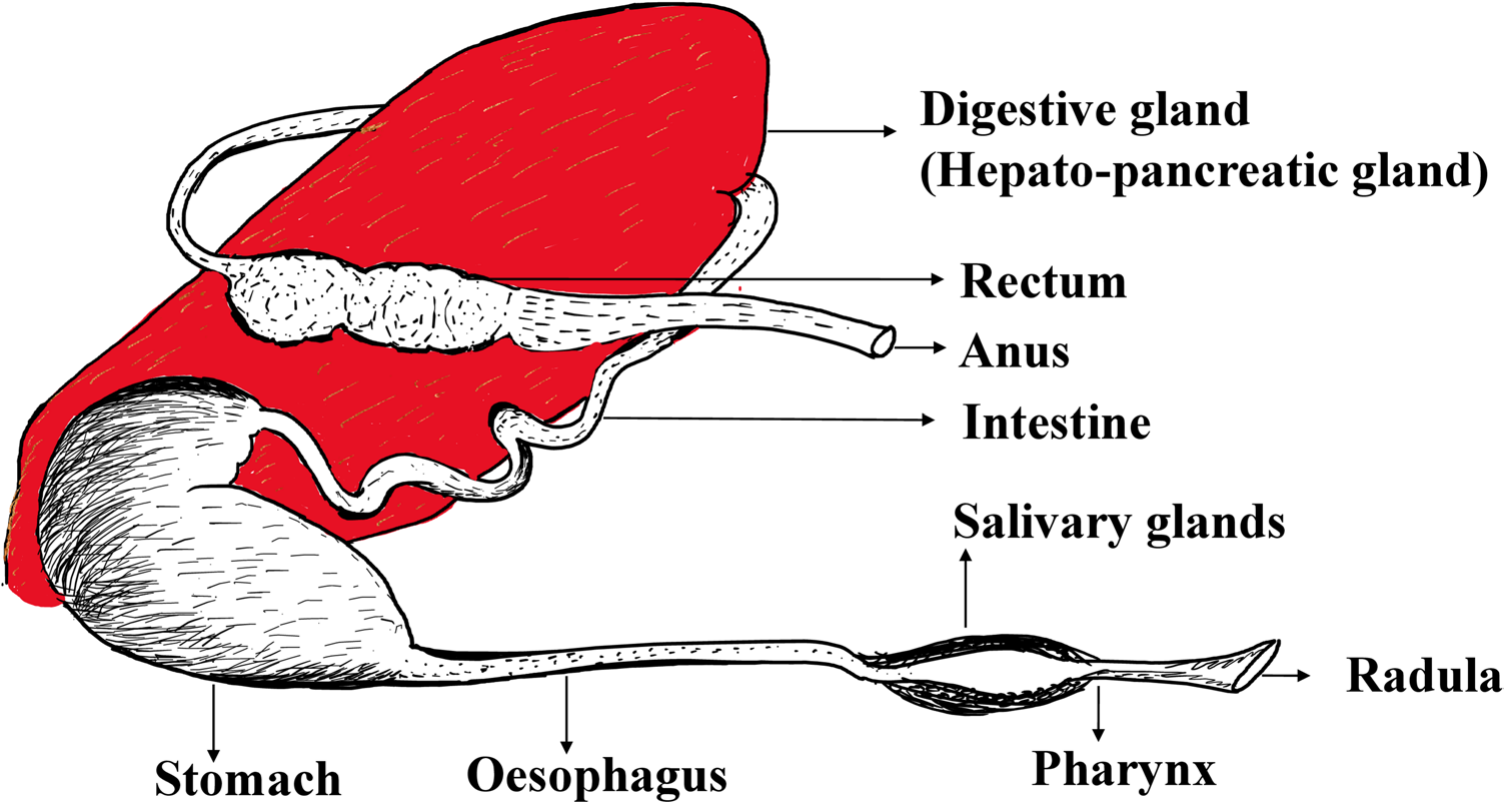
The digestive system contains organs and digestive glands (in red)*.* Adapted and modified from published literature (Ponder et al. 2019b)

Parasites can induce systemic physiological changes and pathological lesions in the different organs of snails. Snails with different natures or feeding habits and life stages are prone to infection with a wide range of pathogens via the oral route through contaminated feeding materials (Marcogliese and Pietrock 2011). Land snails possess an esophagus, and the crop for temporary storage and digestion of ingesta, but freshwater snails have a gizzard, which is a strong muscular stomach that helps in the final digestion of ingesta (Fig. 3) (Ponder et al. 2019a; Pouil et al. 2020). Herbivores possess an oral cavity that employs amylase to process food material and eliminate harmful pathogens, allowing both to pass along with ingesta (Escobar-Correas et al. 2019; GHOSE 1961). Conversely, carnivores lack any amylase activity in their oral cavity. Nutrients derived from the digestion of food, such as amino acids, glucose, and fatty acids, can be utilized by the larval stage of parasites like redia and sporocysts (Malek 2018). Any interferences in the digestion of food, absorption, and metabolism of nutrients lead to the loss of tolerance by snails against environmental stress (Ponder et al. 2019b). Once nutrients are absorbed from the gastrointestinal tract of snails, they enter the circulatory system of snails.

### The circulatory and respiratory systems of snails

**Fig. 4:**
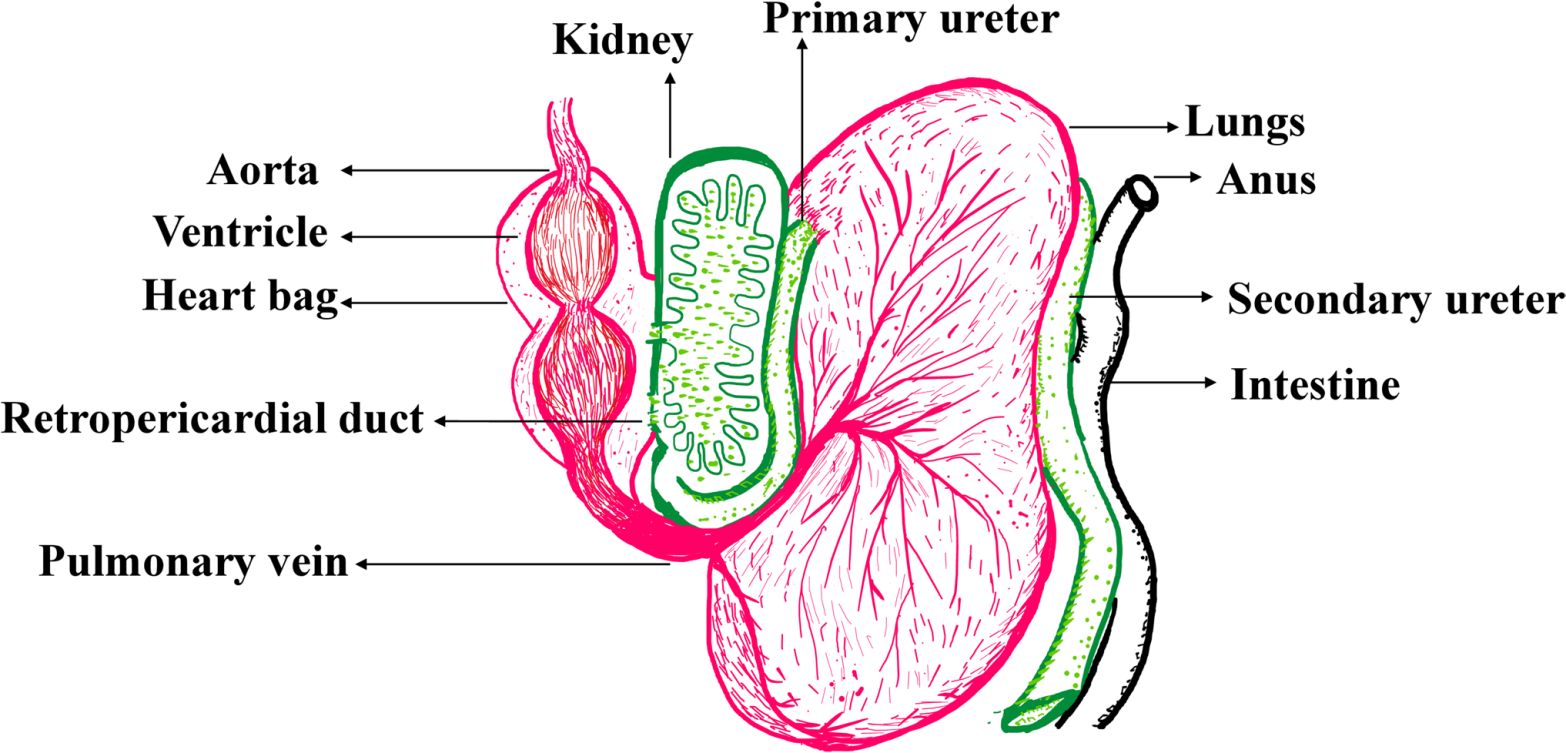
Representing different parts of the excretory in green, circulatory in pink, and respiratory systems in pink of the snail. Adapted and modified from published literature (Ponder et al. 2019b)

Snails have an open circulatory system with a heart bag serving as the central organ for the pumping of haemolymph (blood) into the body cavity of snails (Fig. 4). Within the cavity, haemolymph bathes various organs and tissues (Ponder et al. 2019a). However, the majority of the larval stages of trematodes, like daughter sporocysts and cercariae, migrate posteriorly or to the distal end opposite to the flow of direction of the haemolymph infecting ovotestis and albumen glands (Malek 2018). Such trematode infections in the respiratory organs of snails lead to inflammatory reactions, congestion, and spread to various organs (Blair 2022; Malek 2018). Hence, the visceral organs of snails are prone to parasitic lodgement, infestation, and inflammatory responses. Thus, snails have adapted their lifestyle to efficiently fulfill their growth and reproductive functions in response to parasitic infections.

### The reproductive system of snails

**Fig. 5:**
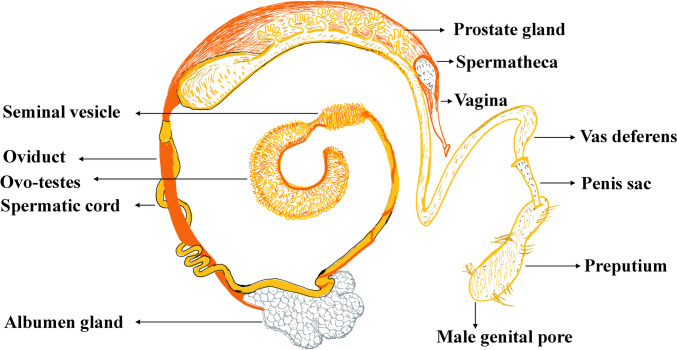
Representing the reproductive system of a snail: male in yellow and female in orange. Adapted and modified from published literature (Ponder et al. 2019b)

**Fig. 6:**
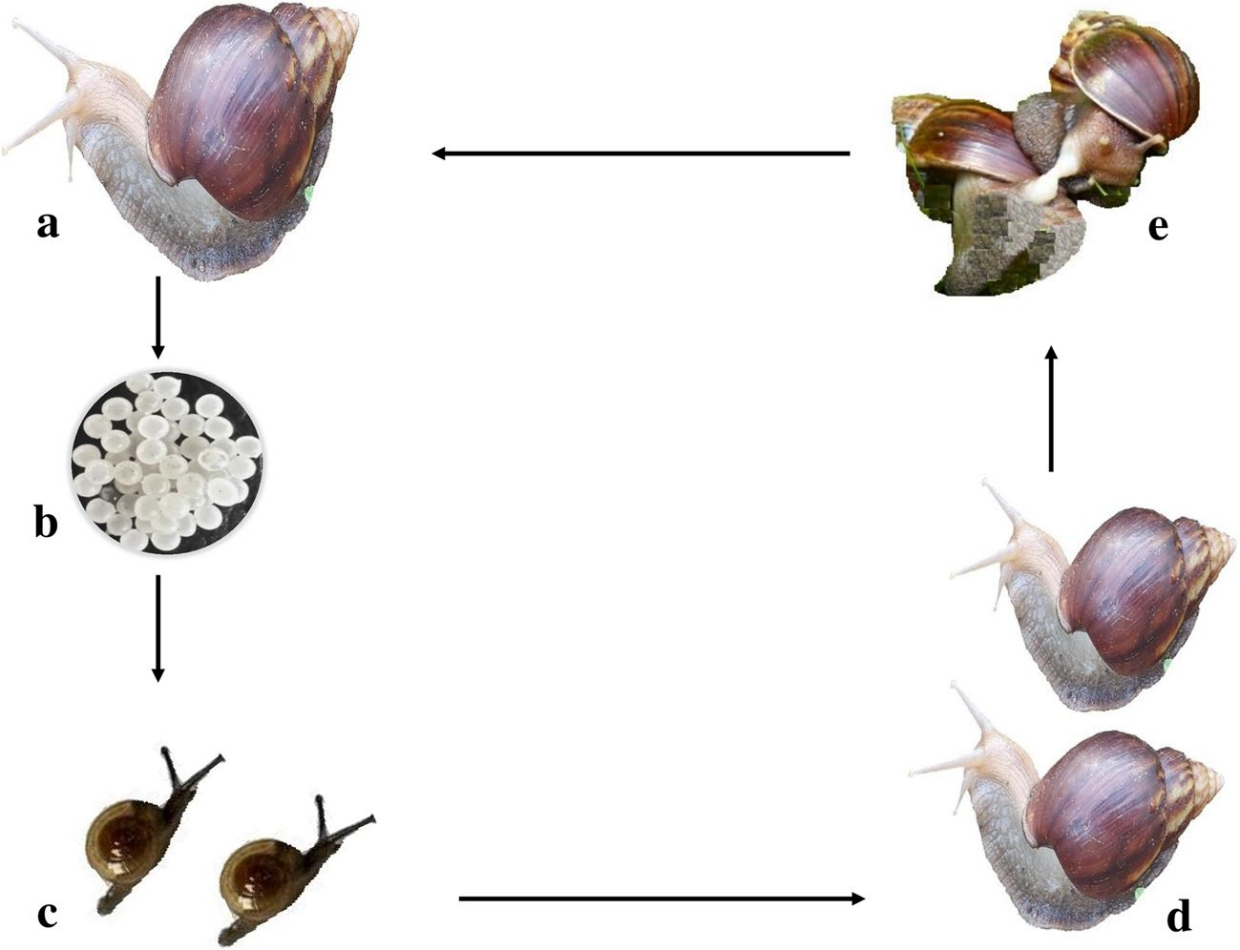
Representing the general life cycle of a snail. **a** Fertilized adult snail. **b** Snails eggs in clusters. **c** Juveniles. **d** Adult. **e** Mating

Most snail species are hermaphrodites, as they contain both male and female reproductive organs (Fig. 5). However, cross-fertilization occurs in snails to fertilize their eggs. After fertilization, snails lay eggs under soil or leaves, and the eggs hatch into the small juvenile stage (Fig. 6) (Ponder et al. 2019b). The reproductive system of snails does not appear to have a direct role in the growth and proliferation of infecting parasites, unlike digestive or circulatory systems. The duration of life stages and the development of body structures of snails can vary depending on environmental factors and their nutrient supply (Erkano 2021). As discussed below, the overall reproductive performance and weight gain of snails are affected by the interplay of biotic and abiotic factors surrounding them.

## Environmental factors affecting snails’ growth and parasitic disease transmission

Environmental conditions lead to changes in both the physiology and morphology of snails. External abiotic factors, such as temperature, salinity, oxygen, and pH, influence the physiological processes of snails (Bula et al. 2017; Donham et al. 2022; Saallah et al. 2020).

### Temperature

Temperature plays a vital role in the physiochemical activities of snails. For example, in *Pila globosa*, higher lipid peroxidation (51.8%) and antioxidative activity (26.4%) were reported in the summer than in other seasons (Pati et al. 2023). During environmental stressful situations, heat shock proteins interact with chaperone-assisted proteins to maintain cellular homeostasis and the survival of land snails, *Helix pomatia* Linnaeus (Idczak-Figiel et al. 2023). During summer, higher physical activities of snails lead to their migration, breeding, and growth. In the freshwater snails (*Bulinus glolosus*), at the temperatures of 15.5 °C and 36.0 °C, snails were unable to produce eggs, but egg mass output was found to be the highest at 25.8 °C (Kalinda et al. 2017). The optimum temperature for snail hosts and their infecting parasites is around 27 °C. The relative abundance of snail parasites can decrease by 8 to 17%, with the rise in temperature above the optimum level by 1 to 2 °C, respectively (Kalinda et al. 2018). These studies indicate that both snails and infecting parasites require a unique temperature range for their interaction, proliferation, and survival (Nguyen et al. 2020).

### Oxygen, salinity, and pH

The abundance of snail species in certain habitats is also determined by their capacity to utilize oxygen either from the air or dissolved in surrounding environments, and this factor is more critical for aquatic snail species. For example, a higher abundance of *Bulinus globosus* (31.7%) followed by *Lymnaea natalensis* (21.6%), *Lymnaea truncatula* (15.1%), and *Biomphalaria pfeifferi* (14.6%) in the Ethiopian Rift Valley region was associated with variations in the oxygen concentrations of water bodies and the oxygen utilizing abilities of snails (Olkeba et al. 2020). Embryonic survival and development of snails were negatively affected by increased environmental salinity even though embryos reside inside their protective egg mass (Barrios-Figueroa and Urbina 2023). The optimum pH for snails is 7.8 to 8.5. Thus, a rise in acidity can lead to disturbances in the enzymatic activity of snails, and their shells may become more transparent and thinner and crack (O'Sullivan et al. 2011).

There is a lack of comprehensive *research* on the cellular signaling pathways of snails in response to variations in environmental factors. Understanding the impacts and interactions of various environmental parameters on the growth and physiology of snails and infecting parasites is critical while aiming to exploit snails for various commercial applications.

## Snail-transmitted parasitic diseases in animals and humans

STPDs are a significant problem in veterinary and public health, causing heavy economic losses. Based on the class of parasites of snails, STPDs can be divided into three groups: trematode diseases (by infection of flukes such as liver flukes, intestinal flukes, blood flukes, and lung flukes), nematode diseases (rat lungworm infection), and cestode diseases (*Davainea* infection). The majority of STPDs are responsible for clinical manifestations and lesions in livestock and humans and belong to digenetic trematodes, metastrongyle nematodes, and a few tapeworms (Table 4) (Lu et al. 2018). As discussed in the following, the dietary habits of humans, animals, and birds can influence the prevalence of snail-associated parasitic diseases.

**Table 4:** Snail transmitted parasitic diseases: parasite species, intermediate and definitive host, parasitic life stages, transmission, and pathological lesions

DISEASE Parasite species	DH	Parasitic stage		Transmission	Pathological lesions	References
		Snail	2^nd^ IH			
FASCIOLOSIS *Fasciola hepatica*, *F. gigantica*	Human Sheep Goat Cattle	**Mi-Sc-R**-**C**	-	Food, water vegetation **Mc** via ingestion	Hepatomegaly, cirrhosis, bile obstruction	Lalor et al. (2021), Mehmood et al. (2017), Sabourin et al. (2018)
SCHISTOSOMIASIS *Schistosoma nasalis*, *S. mansonia*, *S. japonicum*, *S. haematobium*	Cattle Human	**M**i**-Sc**- **C**	-	Skin penetration by **Cercaria**	Dermatitis, granuloma of the nasal cavity, intestinal and urogenital carcinoma	Anderson and Enabulele (2021)
ECHINOSTOMIASIS *Echinostoma* spp.	Birds Human	**Mi-Sc-R-C**	**Mc** in frogs, turtles	Ingestion of raw, undercooked meat (molluscan, crustacean) vegetation	Periportal lymphocytic infiltration and focal hepatic necrosis	Pham et al. (2022), Toledo et al. (2022), Toledo et al. (2019)
PARAGONIMIASIS *Paragonimus westermani*	Cattle Human	**Mi**-**Sp**-**R**-**C**	**Mc** in crabs’, crustaceans	Raw and infected undercooked meat of paratenic hosts.	Pulmonary, neurological, and abdominal lesions	Ahn et al. (2021), Blair (2022)
FASCIOLOPSIS *Fasciolopsis buski*	Pigs Human	**Mi-Sc-R**-**C**	-	Foodborne. **Mc** via ingestion	Intestinal mucosal destruction	Toledo et al. (2019)
CLONOCHIASIS AND OPISTHORCHIASIS *Clonorchis sinensis*, *Opisthorchis felineus*, *O. viverrine*	Dog Cat Human Pigs	**Mi-Sc**-**C**	**Mc** in fish	Foodborne. **Mc** infected fish	Cholangitis, cirrhosis, hepatic fibrosis/cancer	Charoensuk et al. (2022), Fedorova et al. (2020)
EURTYTREMIASIS	Ruminants Herbivores	**Mi-Sc**-**C**	Mc in grasshopper	Ingestion of contaminated vegetation	Interstitial pancreatitis	Schwertz et al. (2015)
ANGIOSTRONGYLIASIS *Angiostrongyliasis cantonensis*	Rat Human	**L1-L2-L3**	-	Ingestion of IH or paratenic host containing **L3**	Human neuro-angiostrongyliasis, Eosinophilic meningitis	Turck et al. (2022)

*DH*, definitive host; *IH*, intermediate host; *Mi*, miracidium; *Sc*, sporocyst; *R*, redia; *C*, cercaria; *Mc*, metacercaria; *L1*, 1^st^ larval stage; *L2*, 2^nd^ larval stage; *L3*, 3^rd^ larval stage

### Fasciolosis

The two species of parasites that cause fasciolosis in humans and animals are *Fasciola hepatica* and *Fasciola gigantica*, commonly known as liver flukes. Fasciolosis due to *F. hepatica* is widely distributed worldwide, whereas *F. gigantica* is found more in Africa, the Middle East, and Asia, with limited distribution of suitable intermediate lymnaeid snail hosts (Mehmood et al. 2017). In the global scenario, fasciolosis has susceptibility to more than 180 million people, whereas more than 35–72 million individuals are considered to be infected with liver fluke (Sabourin et al. 2018). Fasciolosis causes significant economic losses estimated annually at over US$3 billion worldwide (Sabourin et al. 2018).

Liver flukes have a complex life cycle. They involve humans or animals as the definitive host and snails as the intermediate host belonging to the Lymnaeidae family and are responsible for harboring the intermediate stages of liver fluke (miracidium, sporocyst, redia, and cercaria). The ingestion of encysted infective metacercaria stage found on aquatic vegetation is the means of transmission to the final host (Ansari et al. 2021; Caldeira et al. 2016; Hu et al. 2021; Lalor et al. 2021). Preventive measures may include grazing restriction in the areas where snails are abundant or sunlight treatment of grasses from such areas. To prevent human infections, freshwater vegetables should not be consumed raw, because water sources such as artificial irrigation channels are known to harbor snails (Stein 2018). The use of molluscicides or biological means (duck or poultry, plants) can prevent the snail population. One-health approach with multidisciplinary collaborative activities like vaccination of livestock, proper management of freshwater bodies, and improved diagnosis and treatment approaches are crucial steps in mitigating the zoonotic fascioliasis risk (Ali et al. 2017; Mas-Coma et al. 2023). Using triclabendazole to treat human fascioliasis highlights the need to focus on a one-health approach to animal reservoirs for better control (Bargues et al. 2020). Moreover, collaborative efforts from various international organizations, such as the World Health Organization (WHO), the Food and Agriculture Organization (FAO), the World Organization for Animal Health (OIE), and the United Nations Environment Programme (UNEP), could play a pivotal role in controlling and preventing fascioliasis in endemic areas of the world through awareness and education, animal health management, and meat inspection (Corning 2014; Mas-Coma et al. 2023).

### Fasciolopsiasis

Fasciolopsiasis is a foodborne zoonotic parasitic disease caused by the infection of *Fasciolopsis buski* (intestinal fluke) and is transmitted by snails of the Planorbidae family (Lu et al. 2018). Fasciolopsiasis is categorized as a neglected disease by the WHO, and it is considered endemic in specific regions of the Far East and Southeast Asia, especially where pig farming, freshwater habitats, and open-air defecation are common (Siles-Lucas et al. 2021; Toledo et al. 2019). The life cycle of *Fasciolopsis buski* closely resembles that of *Fasciola hepatica* and begins with the hatching of eggs into miracidia, and then the miracidia invade snails of the Planorbidae family (Guerrant et al. 2011; Siles-Lucas et al. 2021). The infected snails, e.g., *Planorbis* spp. and *Segmentina* spp., with miracidia, often feed on plants fertilized with human night soil. The cercariae of *Fasciolopsis buski* then emerge and encyst on the tubers or nuts of these plants, and when humans or animals consume these infected plants and their raw derivatives, it can lead to infection (Siles-Lucas et al. 2021). Infections with *Fasciolopsis buski* are often asymptomatic but can lead to ulcers or abscesses in the duodenum and jejunum, causing epigastric pain resembling peptic ulcer disease (Mas-Coma et al. 2019). Severe cases of fasciolopsiasis may result in eosinophilia, anemia, and systemic allergic symptoms in children (Kimberlin et al. 2022; Long et al. 2012). Preventive measures for both fasciolosis and fasciolopsiasis should include proper sewage disposal, environmental sanitation, education, and personal hygiene (Mas-Coma et al. 2021; Zhou 2022).

### Paragonimiasis

Paragonimiasis occurs in humans and animals by eating raw or inadequately cooked crabs or crayfish infected with metacercaria of *Paragonimus* spp. (Ahn et al. 2021). Several *Paragonimus* spp. have been reported to cause human infections globally. However, these infections are more common in Asian enclaves, including Korea, Japan, China, Taiwan, the Philippines, Thailand, Vietnam, and India (Blair 2019). Species of snail-like *Melanoides* and *Tarebia* act as the first intermediate host (Paller et al. 2021). This disease mainly infects livestock and humans, leading to pathological lesions, most commonly of the pulmonary system, although extra-pulmonary infections in the central nervous system also occur (Ahn et al. 2021). Effective anthelmintic drugs that can be used against paragonimiasis are praziquantel and triclabendazole (Richter 2022). In addition, the application of a one-health approach, such as raising awareness, implementing diagnostics, and emphasizing the importance of preventive measures for parasitic infections, is essential. Moreover, dietary recommendations include avoiding consuming raw or undercooked aquatic meat like freshwater crabs and crayfish and ensuring proper cooking to prevent Paragonimiasis effectively (Farag et al. 2023; Sadhukhan 2022).

### Schistosomiasis

The infection of blood fluke of various species of *Schistosoma* causes schistosomiasis. The WHO has reported that approximately 770 million people are at risk of schistosomiasis, and it is spread over more than 78 countries in Africa, Asia, and Latin America, especially in poor communities (WHO 2022). *Biomphalaria*, *Lanistes*, *Planorbarius*, *Neotricula*, *Oncomelania*, *Robertsiella*, and *Melanoides* species of snails are responsible for acting as intermediate hosts for these blood flukes (Ovando and Marchi 2021; Saito 2022). The infective stage is either schistosomula or cercarial penetration on the skin (Anderson and Enabulele 2021; Li et al. 2021; Limpanont et al. 2020). The use of night soil (human waste) as fertilizers has been applied since ancient times (Kawa et al. 2019), and it has acted as a potential facilitator for parasites excreted by feces. In China, in 2007, half of the farmers in 36 selected villages applied night soil to crops, and its usage was highly associated with *S. japonicum* infections (Carlton et al. 2015). A preventive measure for schistosomiasis could be the breaking of the life cycle of the parasite in the endemic areas. The WHO employs several strategies to combat schistosomiasis. One key method is preventive treatment using the drug praziquantel, given as mass drug administration (MDA) (King et al. 2020). This approach targets high-risk populations, like school-age children and adults living in endemic areas (like Sub-Saharan Africa and Yemen), to reduce schistosomiasis infection and related health problems (Deol et al. 2019). The WHO also conducts health education campaigns to inform communities about the disease’s risks, transmission, and prevention, such as creating awareness toward clean and safe water sources in endemic regions. In non-endemic areas (like Europe and North America), strategies have focused on targeting the risks and safety measures for imported cases (Gabrielli and Garba Djirmay 2023). Surveillance systems help monitor disease prevalence and program impact while research improves diagnostics and treatment methods. Such integrated one-health approaches ultimately play a significant role in reducing the burden of schistosomiasis worldwide.

### Echinostomiasis

Echinostomiasis is one of the most neglected trematode infections in humans (Toledo et al. 2019). Humans become infected after ingesting raw or insufficiently cooked molluscs, fish, crustaceans, amphibians, or aquatic vegetables (Toledo et al. 2022). Echinostomiasis is endemic in Southeast Asia, the Middle East, and East Africa (Siles-Lucas et al. 2021). Adult *Echinostoma* spp. of parasites commonly inhabit the intestines of a wide range of birds and mammals, including humans (also recorded in reptiles and fishes) (Pham et al. 2022; Seo et al. 2020; Toledo et al. 2019)*.* The prevalence of echinostomiasis in endemic areas, especially in Sub-Saharan African and Asian countries, remained unchanged despite environmental measures and education (Yigezu et al. 2018). Cercarial infection rates of *Echinostome* spp. in snail species decreased from 58 to 3.6% between 2007 and 2016 in South-West Ethiopia, which could be due to increased resistance development by snails against the parasitic load (Mengistu et al. 2011; Mereta et al. 2019; Toledo and Esteban 2016). Protecting fishponds from night soil fertilization or fecal contamination, proper waste management, and education campaigns are important measures to protect potential hosts from this parasitic infection (Toledo and Esteban 2016).

### Clonorchiasis and opisthorchiasis

Clonorchiasis and opisthorchiasis are parasitic diseases caused by the infections of *Clonorchis sinensis*, *Opisthorchis viverrini*, and *Opisthorchis felineus* flukes (Pozio and Morales 2022). These parasitic diseases are major public health problems affecting over 15 million in the trans-national region of the Mekong River basin in Asian countries: Cambodia, Yunnan Province and Guangxi Zhuang of China, Lao, Myanmar, Thailand, and Vietnam (Sripa et al. 2021). Snails of the Bithyniidae family have a major role in the transmission cycle as the first intermediate host, while fish act as the secondary/paratenic host (Charoensuk et al. 2022; Ovando and Marchi 2021). There should be proper selection, handling, and treatment of food of aquatic origin before consumption, as consumption of undercooked fish is the main route of infection in humans. Education, improving sanitary conditions, and treatment can be effective in preventing infections, especially in areas where human night soil and pig feces have been applied as nourishment for the fish.

### Angiostrongyliasis

Angiostrongyliasis has been reported increasingly in parts of Asia, the Pacific islands, Australia, the USA, the Caribbean (initially Cuba), the Caribbean islands, Mallorca, and the Canary Islands. It is now widely distributed across many tropical/sub-tropical and a few more temperate localities, with the parasite found in numerous host species (Turck et al. 2022). It is estimated that more than 7000 cases of neuro-angiostrongyliasis have been reported in humans (Niebuhr et al. 2019). *Angiostrongylus cantonensis* (rat lungworm), the causative agent for angiostrongyliasis, is a nematode parasite having a complex life cycle where rats are the final hosts, gastropods (snails) act as intermediate hosts, and with several paratenic hosts (freshwater prawns/shrimp, crabs, flatworms, fish, frogs, toads, lizards, and centipedes) (Turck et al. 2022). The wide range of snail species (more than 60) belonging to gastropods is responsible for harboring the intermediate larval stage of this nematode (Brozzo et al. 2020; Segeritz et al. 2021; Turck et al. 2022). Since the wide range of infections occurs in different snail species and humans and other animals, it is crucial to raise awareness of the associated dangers of consuming snails as food and feeding materials. These food preferences are regarded as customs in, for example, China and Thailand, where undercooked snails have been consumed for generations (Wang et al. 2012). Changing such preferences may be challenging regardless of educational efforts. In Thailand, studies have shown that *Angiostrongylus* parasites, responsible for human angiostrongyliasis, were present in up to 33.3% of snail species, including *Pila virescens* and *Pomacea canaliculata* (Watthanakulpanich et al. 2021). For better prevention and control of angiostrongyliasis, regular screening and monitoring of its associated parasite in food, meat, and environment are needed, potentially applying one-health principles for the presence of the infective parasite (Sears et al. 2021; Turck et al. 2022).

## Application of diagnostic techniques to detect the STPDs

The successful application of diagnostic techniques or tools for detecting the presence of parasites in snails, environment/habitats (water, soil), paratenic hosts, and the definitive hosts requires accurate knowledge of species of parasites and their snail vectors. Such techniques should be rapid, sensitive, and robust for detecting and identifying different stages of parasites (Hammoud et al. 2022). These diagnostic tools are crucial for adequately managing STPDs, limiting their spread from the environment to snails, humans, and animals (Table 5).

**Table 5:** An overview of multiple diagnostic techniques to detect snail-transmitted parasites

Diagnostic technique types	Diagnostic principle	Advantages	Disadvantages	References
Direct shedding	Microscopy and culture-based	• Examination of snail tissues, body fluid, and shedding material • Relatively cheaper	• Need expert in malacology and parasitology	Joof et al. (2020)
ELISA and ICA	Serology-based Ag-Ab reactions	• Sensitive and more specific than conventional approaches • Best for disease screening in endemic areas	• Sometimes, cross-reactivity can occur	Yuan et al. (2023)
Conventional PCR	Genetic material amplification and detection	• Possible to detect DNA or RNA from both parasites and snail hosts • Confirmatory test	• Needs an equipped laboratory • More expensive compared to microscopy and serology	Rathinasamy et al. (2021)
qPCR coupled with eDNA	PCR and genomics	• Highly sensitive to the detection of tiny amounts of organism genes from soil and water • More precise and can cover a broader habitat than conventional PCR	• Needs an equipped lab • Can be more expensive compared to conventional PCR, serology	Rathinasamy et al. (2021)
LAMP	Amplification of genetic material	• Simple and rapid • Assist to improve current surveillance techniques (like eDNA)	• False-positive result due to primer-primer reaction and contamination	Tran et al. (2022)

*ELISA*, enzyme-linked immunosorbent assay; *ICA*, immunochromatographic assays: *PCR*, polymerase chain reaction: *qPCR*, quantitative polymerase chain reaction; *eDNA*, environmental DNA; *LAMP*, loop-mediated isothermal amplification; *Ag*, antigen; *Ab*, antibody

Direct shedding and microscopy are the traditional techniques used for the detection/identification of parasites in snails, where the snail tissues and their shedding materials are examined under a microscope to visualize the parasites (Joof et al. 2020). Serology-based techniques, for example, enzyme-linked immunosorbent assay (ELISA) and immunochromatographic assays (ICA), can be sensitive and specific for detecting parasitic antigens in snail tissues or body fluids (Yuan et al. 2023). The polymerase chain reaction (PCR) techniques were found to be more sensitive than the conventional shedding techniques for the detection of infection of *S. mansoni* in the *Biomphalaria* species of snails (Joof et al. 2020).

Multiplex PCR (simultaneous detection of multiple species of parasites and snails) and qPCR (amenable to high-throughput and multiplexing, sensitive, faster, cheaper) are methods of choice coupled with environmental DNA (eDNA). The eDNA detection technique can be a potential tool for monitoring of parasitic agents in water/soil samples (Jones et al. 2018). The limit of detection of the eDNA assays for the detection of *Fasciola hepatica* and snails (*Austropeplea tomentosa*) was observed to be 14 fg and 50 fg genomic DNA, respectively, in water samples (Rathinasamy et al. 2021). Thus, such eDNA-based techniques can be useful in detecting snail-borne parasites in various environmental samples (Rathinasamy et al. 2021).

Loop-mediated isothermal amplification (LAMP) is another molecular technique that is simple and rapid and can be used for the detection of parasites in snails (Tran et al. 2022). Recently, it has been suggested that LAMP results for certain STPDs, such as schistosomiasis, appear to be comparable to or better than commonly used diagnostic techniques (García-Bernalt Diego et al. 2021). PCR is a widely used molecular technique used for the detection of DNA or RNA either from parasites or from snails.

## Future perspectives

**Fig. 7:**
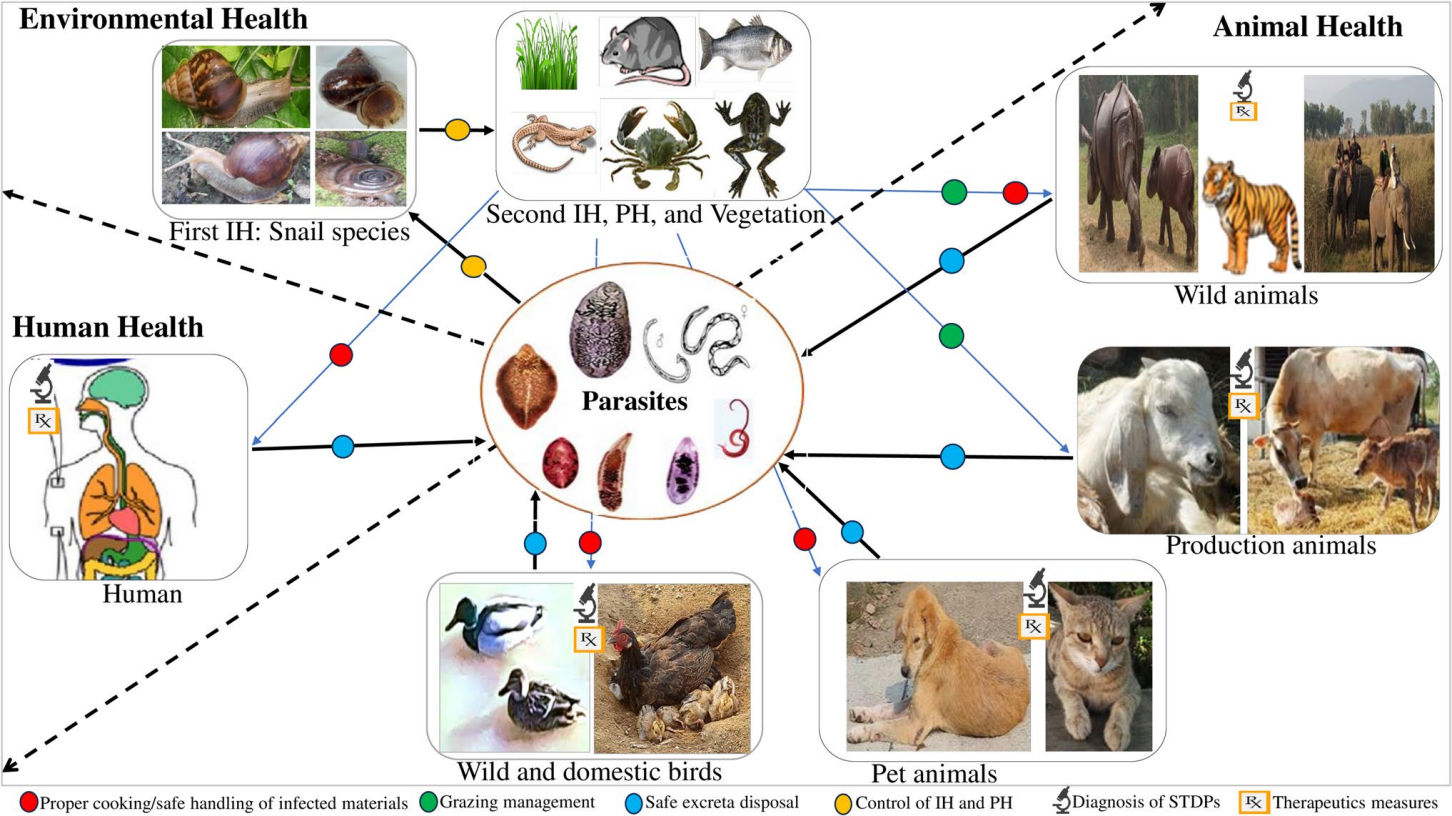
Demonstrating the multiple aspects of a one-health approach for STPDs. IH, intermediate host; PH, paratenic host

It is noteworthy that STPDs are not only a concern of public health, but they also have the potential to be cross-transmitted between animals and birds. STPDs, like schistosomiasis, fascioliasis, and angiostrongyliasis, pose a significant threat to human and animal health globally (Hammoud et al. 2022). In the future, proper prevention and control of STPDs appears to be a significant challenge for public health systems and veterinary medicine. The one-health approach to most STPDs requires broader collaborative efforts between human and animal health professionals, environmental experts, and public health authorities. Promoting surveillance, health education, veterinary care, and environmental management can mitigate the risks and minimize the transmission of these STPDs. With advancements in diagnostic and treatment methods, there is hope for better management of these diseases, especially with the application of one-health approaches (Fig. 7).

The dynamics of STPDs are likely to be affected by several factors, climate change, modified temperature, and rainfall patterns, which can affect the distribution and abundance of snail species and, consequently, the transmission of the diseases (Bula et al. 2017; Donham et al. 2022; Saallah et al. 2020). Likewise, urbanization and globalization can facilitate the spread of snails and parasites across borders and increase human exposure through travel and trade (Celis-Ramírez et al. 2022). While snails can serve as hosts for disease-causing parasites, it is important to note that they also play important roles in ecosystems as food sources for other animals and as decomposers. Therefore, any efforts to control snail populations and STPDs should be implemented in a naturally responsible manner and considered in conjunction with the current epidemiological situation in the endemic areas.

Snails are high in protein content, making them a nutritious food source for livestock and other animals (Uboh et al. 2014). Moreover, they efficiently convert feed into biomass, requiring fewer resources than traditional livestock (Mvodo Meyo et al. 2021). However, it is noted that snails can serve as hosts for disease-causing parasites, and there is a risk that these parasites could be transmitted to animals consuming snail-based feed. In addition, if snails are not raised under proper conditions, they may accumulate harmful substances in their tissues that could be toxic to animals (Bula et al. 2017; Donham et al. 2022; Saallah et al. 2020). Therefore, the establishment of appropriate protocols and methods for snail farming is essential to producing safe and nutritious snails that lead to minimal risks associated with the spread of STPDs. Moreover, strict regulations and monitoring should be put in place to ensure that snails used for animal feed are free from harmful substances and parasites.

Looking forward, the potential of snails as an alternative feed source should be explored further while ensuring that any hazards associated with their use are minimized. With careful planning and management, snails have the potential to be commercially utilized as food or feed sources that are sustainable, safe, and nutritious for humans and animals.

## Conclusions

Snails are natural scavengers and play a vital role in the ecosystem. Both land and water snail species are prone to be infected with parasites from their surrounding environments. The physiological systems and visceral organs of snails are prone to parasitic lodgement, infestation, and inflammatory responses, and such parasitic infections in snails can compromise the growth and development of snails. As snails appear to be increasingly exploited for food or feed applications in the future, parasites invading snails can transmit to humans and animals. Such transmission can increase the risks of various parasitic diseases in humans and animals, including but not limited to fasciolosis, fasciolopsiasis, schistosomiasis, and angiostrongyliasis. These STPDs pose significant threats to human and animal health globally. Understanding the impacts and interactions of various abiotic factors on the growth and physiology of snails and infecting parasites is critical while aiming to exploit snails for various commercial applications. In this context, the application of a one-health concept is necessary to maximize the commercial utilization of snails as food and feed while minimizing the risks of snail-associated parasitic diseases. Various diagnostic tools can assist in detecting snail-infecting parasites in food, feed, animals, and the environment. The integrated use of more sensitive, specific, and cheaper diagnostic tools allows simultaneous monitoring of disease prevalence for better control and prevention of STPDs. Moreover, appropriate farming methods and protocols for snails must be developed, and regulations should be put in place to minimize the associated hazards of using snails as an alternative nutrient source. As the potential of snails as an alternative source of feed for animals is promising, future research efforts are necessary to ensure the safe and responsible use of snails for the benefit of human, animal, and environmental health.

## Data Availability

Not applicable.

## Abbreviations

AC: Angiostrongylus *cantonensis*
C: Cercaria
Ca: Calcium
CF: Crude fiber
CHO: Carbohydrates
CS: *Clonorchis sinensis*
DH: Definitive host
IH: Intermediate host
FB: *Fasciolopsis buski*
Fe: Iron
DM: Dry matter
FG: *Fasciola gigantica*
FH: *Fasciola hepatica*
L1: 1^st^ larval stage
L2: 2^nd^ larval stage
L3: 3^rd^ larval stage
Mc: Metacercaria
Mi: Miracidium
OF: *Opisthorchis felineus*
OV: *Opisthorchis viverrini*
PW: *Paragonimus westermani*
R: Redia
Sc: Sporocyst
SH: *Schistosoma haematobium*
SJ: *Schistosoma japonicum*
SM: *Schistosoma mansoni*
SMa: *Schistosoma malayensis*
SMe: *Schistosoma mekongi*
STPDs: Snail transmitted parasitic diseases
TP: Total protein
